# Development of a LC-MS/MS Method for the Multi-Mycotoxin Determination in Composite Cereal-Based Samples

**DOI:** 10.3390/toxins9050169

**Published:** 2017-05-18

**Authors:** Barbara De Santis, Francesca Debegnach, Emanuela Gregori, Simona Russo, Francesca Marchegiani, Gabriele Moracci, Carlo Brera

**Affiliations:** GMO and Mycotoxin Unit, Food Safety, Nutrition and Veterinary Public Health Department, Istituto Superiore di Sanità, Viale Regina Elena 299, Rome 00161, Italy; francesca.debegnach@iss.it (F.D.); emanuela.gregori@iss.it (E.G.); simon_says_go@hotmail.it (S.R.); francesca.marchegiani@libero.it (F.M.); gabriele.moracci@iss.it (G.M.); carlo.brera@iss.it (C.B.)

**Keywords:** mycotoxin, aflatoxins, ochratoxin A, deoxynivalenol, fumonisins, T-2 and HT-2 toxins, zearalenone, Total Diet Study, liquid chromatography-tandem mass spectrometry

## Abstract

The analytical scenario for determining contaminants in the food and feed sector is constantly prompted by the progress and improvement of knowledge and expertise of researchers and by the technical innovation of the instrumentation available. Mycotoxins are agricultural contaminants of fungal origin occurring at all latitudes worldwide and being characterized by acute and chronic effects on human health and animal wellness, depending on the species sensitivity. The major mycotoxins of food concern are aflatoxin B1 and ochratoxin A, the first for its toxicity, and the second for its recurrent occurrence. However, the European legislation sets maximum limits for mycotoxins, such as aflatoxin B1, ochratoxin A, deoxynivalenol, fumonisins, and zearalenone, and indicative limits for T-2 and HT-2 toxins. Due to the actual probability that co-occurring mycotoxins are present in a food or feed product, nowadays, the availability of reliable, sensitive, and versatile multi-mycotoxin methods is assuming a relevant importance. Due to the wide range of matrices susceptible to mycotoxin contamination and the possible co-occurrence, a multi-mycotoxin and multi-matrix method was validated in liquid chromatography-tandem mass spectrometry (LC-MS/MS) with the purpose to overcome specific matrix effects and analyze complex cereal-based samples within the Italian Total Diet Study project.

## 1. Introduction

As is known, the change of climatic conditions of the planet will determine a warming of the eco-system leading to an unavoidable increase of the probability of the occurrence of fungal attack and mycotoxin production in the majority of crops worldwide [1,2,3,4,5]. The more immediate fallout is the increase of the menace of a further limitation of food availability prejudicing food security firstly and food safety secondly, as recently reported for the most dangerous hazard among mycotoxins, namely aflatoxin B1 [6]. Since the entire agri-food system is involved in this challenge, any stakeholder belonging to any position and role must deserve the highest attention in encouraging the adoption of preventive actions aimed at minimizing the phenomenon as much as possible; this, in view of guaranteeing the availability of safe feed and food products to the final consumer. In addition, the inherent health implications due to the consumption of mycotoxin-contaminated feed and food products pose a real alarm both for animals wellness, with concomitant consequences for the productive yields, the economic gain and the quality of processed foods and the consumer health with a direct impact towards the more sensitive consumer groups such as infants, children, and adolescents together with other situations regarding specific status such as the pregnancy and people affected by coexistent pathologies such as celiac disease.

These aspects are even more relevant in consideration of the probability to have the concomitant presence of more than a mycotoxin in a feed or food product [7,8] that can pose a real, and still now underestimated, increased level of risk for the end-consumer due to any potential additive, antagonistic, or synergistic toxic effects. Recently, a combined toxicity of deoxynivalenol (DON) and fumonisins (FBs), or aflatoxins (AFs) and fumonisin B1 (FB1), in the livers of piglets caused higher histopathological lesions and immune suppression [9,10]. Lee and Ryu summarized the most relevant studies reporting additive or synergistic effects due to the co-occurrence of mycotoxins and their interactive toxicity [11]. Severe reductions in growth and immune response were found in broilers by dietary combinations of AFs and ochratoxin A (OTA) [12].

For the abovementioned reasons, the need of the availability of accurate precise and sensitive analytical methods able to detect the mixture of mycotoxins in a reliable way, plays a pivotal role both for toxicological and exposure assessment issues.

To date, maximum levels (MLs) have been set for the majority of countries worldwide in different food products for different individual mycotoxins with recognized adverse effects, but a new scenario could be depicted in the near future, by the reconsideration of these levels in light of the proved increase of the toxicity due to the co-presence of mycotoxins in comparison with the one derived from the individual toxins. Furthermore, all of the Health-Based Guidance Values (HBGV) have been established for individual toxins and not for their forms of mixture.

Another new aspect to be considered is related to the co-presence of the well-known and studied mycotoxins with the so-called emerging toxins, such as enniatins and T-2 and HT-2 toxins, even if, in a recent review from EFSA, the European Authority of Food Safety, no conclusion on risk assessment for the co-presence of enniatins and beauvericin was possible due to the paucity of data available in feeds and food products.

Hence, it is important to fill the gap by carrying out validated analytical tools aimed at obtaining the accurate assessment of human and animal exposure to this group of mycotoxins by determining their levels in feeds and foodstuffs.

For the determination and quantification of mycotoxins in complex matrices, analytical methods based on liquid chromatography-tandem mass spectrometry (LC-MS/MS) have been extensively used [13,14,15,16]. With the aim to reduce the matrix effects as much as possible by reducing the interferences from the extraction step, a wide variety of sample preparations, such as liquid-liquid extraction, solid phase extraction (SPE), accelerated solvent extraction (ASE), matrix solid-phase dispersion, and dilute and shoot approaches, have been reported [17].

In a recent review [18], it was outlined that, as far as multi-mycotoxin methods, despite the advantages of these multi-mycotoxin methods with respect to conventional methods due to the superior specificity, sensitivity, and fast data acquisition features, which allow simplified sample preparation, improvements in accuracy and efficiency, as well as in the management of matrix effects are still needed [19].

As known, the matrix effects represent one of the most challenging issues, as well LOQ value, to be solved depending on the final endpoint of the analytical research. It should be underlined that multi-mycotoxin analyses must deserve high flexibility from the researchers’ point of view in order to choose the more suitable approach to match the performance characteristics with the different targeted use [18].

This aspect is even more crucial if the composition and the physical nature of the sample to be analyzed corresponds to a quite high complexity of the matrix as the one investigated in a total diet study (TDS) for which the proposed analytical methodology has been set up. The cited TDS, “The Italian Total Diet Study 2012–2014” that included mycotoxins due to their high toxicity and wide frequency in the dietary foods, was organized for the first time in Italy with the aim to develop and spread the TDS methodology on the basis of harmonized principles in terms of study design, sampling and exposure assessment [20,21].

From the above, within the implementation of the Italian TDS, a versatile LC-MS/MS multi-mycotoxin and multi-matrix method was validated with the purpose to guarantee a reliable analysis of cereal-based samples characterized by high sensitivity and applicability.

## 2. Results and Discussion

### 2.1. LC-MS/MS Optimization

The optimization of the LC-MS/MS parameters was conducted by directly applying tuning solutions of the selected mycotoxins. Mycotoxin determination was performed in positive ionization mode after testing the negative ionization mode, especially for DON and ZEA evaluation. Formic acid and ammonium formate were added to facilitate the formation of the protonated precursor ion or the ammonium adduct. Only for T-2 toxin, the sodium adduct, usually considered not suitable, was selected as precursor ion. Tests using different concentrations of HCOOH/NH_4_COOH and adding acid and ammonium salt only to the aqueous component of the mobile phase were performed. The highest peak intensity was obtained by adding the formate buffer to both the eluents at a concentration of 0.3% of HCOOH and 5 mM of NH_4_COOH. Different injection volumes were also tested and, finally, a 10 µL volume was chosen due to a remarkable improvement of the performance in terms of repeatability, 10 µL being the total loop injection mode. Both acetonitrile and methanol were tested as organic modifiers for the mobile phase composition and since no dramatic difference was evidenced, methanol was preferred because it is more environmental friendly in view of laboratory waste disposal.

For identification purpose, the two most intense transitions of the parent compound were selected, while for mycotoxin quantification only the most intense peak (quantifier) was used. In addition, retention time (RT) and ion ratio (IR) variations measured in the samples met the requested criteria (±0.1 min for RT and ±30% for IR), when compared with the value obtained for the calibration standard. In Table 1, precursor and product ions of the tested mycotoxins and the specific MS/MS parameters are presented.

**Figure 1:**
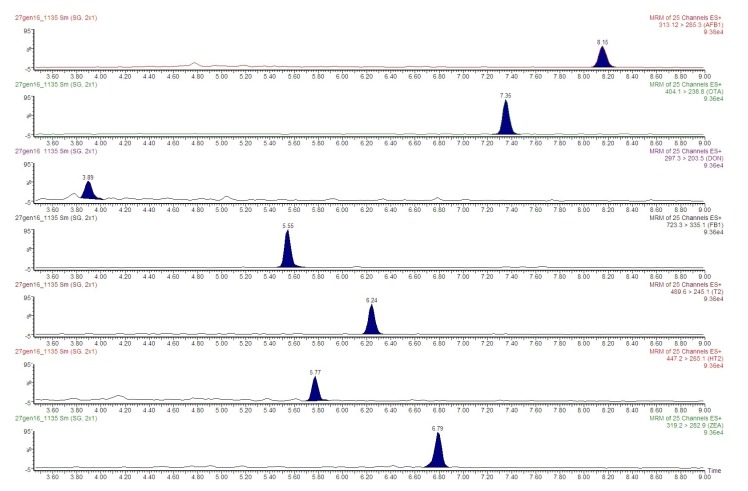
UPLC-MS/MS chromatograms for the considered mycotoxins in the model matrix “Wheat, other cereals and flours” fortified at 0.4 μg/kg for AFB1, 2.5 μg/kg for OTA, 125 μg/kg for DON, 50 μg/kg for FB1, 25 μg/kg for T-2, 125 μg/kg for HT-2, and 10 μg/kg for ZEA.

In Figure 1, chromatograms obtained for the investigated mycotoxins in a fortified sample belonging to the model matrix “Wheat other cereals and flours” are shown.

### 2.2. Optimization of the Extraction Step

LC-MS/MS methods for mycotoxin determination in food samples are often developed with the aim to reduce sample treatment prior to the injection step. Dilute and shoot is a strategy frequently applied since it allows a reduction in time and cost of the analysis and to retain groups of mycotoxins with high chemical diversity. However, the simple dilution of the sample extract may result in an increased limit of quantification (LoQ); on the other hand, the concentration of the sample can lead to strong matrix effect seriously affecting the performance of the method, if no clean-up is applied. The use of isotope dilution is also a strategy to overcome the matrix effect but the entailed costs and availability of isotopologue standards are a real inconvenience to take into account. Thus, in order to avoid sample treatment and to reduce the cost of the analysis, the matrix-matched approach was used. Different extraction mixtures were tested paying attention to the extraction efficiency of the mycotoxins from the composite cereal based food samples and to the co-extraction of interfering compounds that could reasonably result in disturbing matrix effects. In particular, three solvent mixtures, selected from the literature, were tested, namely CH_3_OH:H_2_O 80:20 [22]; CH_3_CN:H_2_O:CH_3_COOH 79:20:1 [15], and EtOH:H_2_O 2:1 [23]; the evaluation of the performance was made on six replicates of spiked blank samples, comparing the recovery factor values and the associated relative standard deviation of repeatability (RSD_r_) obtained. The matrix used for optimizing the extraction phase consisted of pooled extra material obtained from the preparation of the cereal based TDS samples (see Section 3.2). The two first extraction approaches were performed by shaking the sample (1 g) for 30 min with the solvent mixture. After extraction, the samples were treated as described in Section 3.3. The third tested extraction was conducted as reported by Breidbach et al. [23], firstly vortexing the sample with water and then extracting with EtOH in a wrist shaker for 30 min. Magnesium sulfate was added for the salting out effect and, after centrifugation, an aliquot of the supernatant was dried and redissolved with the injection solvent for LC-MS/MS analysis. The obtained results, in terms of recovery factors and RSD_r_, are reported in Table 2. Accordingly, with the data presented in Table 2, the extraction with the mixture with acidified water/organic solvents, namely CH_3_CN:H_2_O:CH_3_COOH 79:20:1, was finally selected. Additionally, the ratio between the weighed sample and the amount of extraction solvent was evaluated. The small amount of sample weighted for the analysis (1 g) was not considered a critical factor in terms of representativity because, in this specific case, the TDS samples originated from thoroughly mixed and processed preparations (see Section 3.2), bypassing the issue of the heterogeneous distribution of the mycotoxins in the matrix. With respect to the amount of the extraction solvent, different quantities were tested; the best results were obtained for the weighted ratio sample:extraction solvent 1:8. Taking into account the nine subcategories to be analyzed for the TDS purpose, six model matrices were chosen as representative of the validation. On the basis of the processing and grain ingredients, the validation was performed on a selected number of model matrices, more specifically, pasta was chosen as a model for the analysis of pasta and pizza samples; bakery products were chosen as a model matrix for biscuits, savory fine bakery products, cakes, and sweet snacks; finally, breakfast cereals, bread, rice, wheat, and other cereals and flours were the remaining matrices used for validation.

### 2.3. Method Performance

#### 2.3.1. Linearity

Linearity was tested by the evaluation of determination coefficients *(R*^2^). The linear range was estimated for curves prepared in neat solvent, as well as for spiked extracts and spiked sample in Table 3. The target value for acceptability of the curve was a *R*^2^ > 0.99, while the residuals were all matrix-matched calibration curves prepared for the six analyzed matrices. Results for R^2^ are reported visually checked to be randomly distributed. As shown from the data reported in Table 3, satisfactory *R*^2^ values were obtained for all the targeted mycotoxins in all the tested matrices.

#### 2.3.2. Apparent Recovery, Matrix Effect, and Extraction Recovery

Results obtained from the injection of calibration curves prepared in neat solvent, spiked extract and spiked samples are presented as apparent recovery (R_A_), signal suppression/enhancement (SSE), and extraction recovery (R_E_), in Table 4. The value of RSD_r_ calculated for R_A_ is shown in Table 4. Since calibration curves were prepared and injected in replicates (*n* = 6) for validation purposes, the LoQ for each mycotoxin, in all of the validated matrices, was assessed as the first point of the spiked extract calibration curve. The validated LoQs are reported in Table 4 for each tested model matrix.

**Figure 2:**
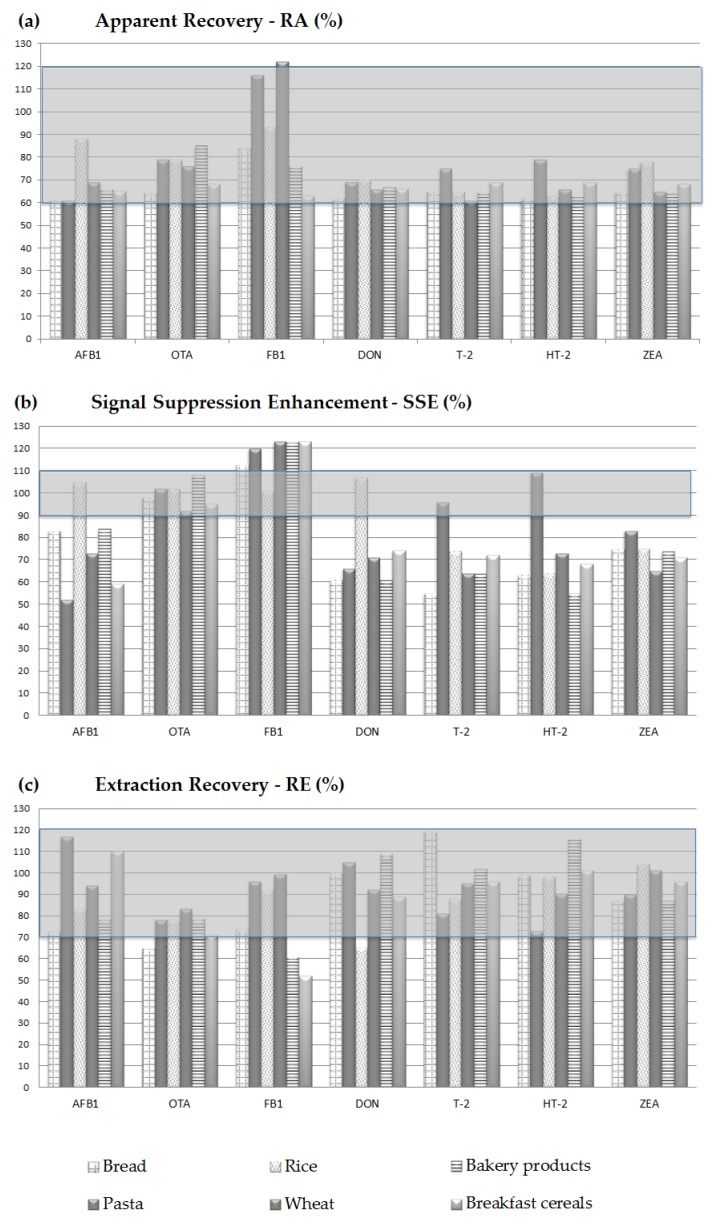
Histograms of the apparent recovery, signal suppression/enhancement, and extraction recovery obtained for all the selected mycotoxins and for each validated matrix. The grey stripe highlights the range 60–120% in (**a**); 90–120% in (**b**); and the range 70–120% in (**c**).

In order to obtain very low LoQ values, a concentration of the sample was applied; as a consequence an increase of the matrix effect was observed, as shown from the SSE values reported in Table 4. The influence of the matrix on the sample ionization depends on the mycotoxin and on the matrix components co-extracted during the analysis. For better evaluation of the influence of the matrix effect, SSEs for different mycotoxin/matrix combinations are reported in Figure 2.

The depicted scenario of SSEs is quite satisfactory, being that the effect of the matrix on the mycotoxin signal is in the range of 60–90% or 110–120% for the majority of the analyzed mycotoxin/matrix combinations. SSE values in these ranges were considered acceptable provided that a matrix match approach is used for the correction of the response. Moreover, the 24% of the tested combinations showed a SSE value in the range 90–110%; in this range SSE may be considered as not affected by matrix effects, according to Malachová et al. [24]. On the other hand, seven out of 42 tested mycotoxin/matrix combinations were below 60% (four combinations) or above 120% (three combinations). However, despite these unfavorable SSE cases, the good performances, in terms of R_A_, R_E_, and RSD_r_, shown in Table 4, support the method reliability when critical values of SSE were also observed.

As for recovery and precision, Annex II of the Commission Regulation (EC) No 401/2006 [25] establishes the performance criteria to which a method shall comply. All of the recovery experiments performed at the LoQ levels for all of the model matrices gave results in the range of acceptability for the tested mycotoxins (namely: >50–120% for OTA and AFB1; 60–110% for DON; 60–120% for ZEA and FBs; and 60–130% for T-2 and HT-2). The calculated relative standard deviations of repeatability were all below 22%, thus confirming a satisfactory performance of method precision even though occasionally a suppression (e.g., AFB1 in pasta or breakfast cereals, T-2 in bread, and HT-2 in bakery products) or enhancement (e.g., FB1 in pasta, wheat, bakery products, and breakfast cereals) was registered.

#### 2.3.3. Application to TDS Samples

The validated method was applied to the analysis of 36 pooled samples obtained within the 2012–2014 Italian Total Diet Study [20]. Examining the levels of contamination of the pooled samples, the 25% resulted lower than LoQ, for all the investigated mycotoxins, the 47% of the samples were contaminated with only one mycotoxin and the 28% of the samples were positive to two or more mycotoxins. In relation to the presence of one or more mycotoxins, DON and ZEA were the most frequently found mycotoxins. Estimated concentrations of DON ranged from LoQ to a maximum value of 200 µg/kg in pooled samples of the “Wheat, other cereals, and flours” subcategory. ZEA was determined at concentrations between LoQ and 40 µg/kg, but no toxin was found in “pasta”, “rice”, “biscuits”, and “savory fine bakery products”.

Moreover, the co-occurring DON + ZEA + FB1 was found in two samples out of 36 of the “Wheat, other cereals, and flours” subcategory.

It is noteworthy to underline that only one pooled sample exceeded the EU maximum levels, namely a sample of subcategory “bread” where the OTA contamination was 18.7 µg/kg.

Finally, considering the high toxicity of aflatoxin B1, it is remarkable that only one sample was positive at a concentration level close to the LoQ value.

## 3. Materials and Methods

### 3.1. Chemicals and Reagents

Methanol (MeOH, Chromasolv^®^ for HPLC, ≥99.9%), acetonitrile (AcCN Chromasolv^®^ for HPLC, ≥99.9%), ammonium formate, and formic acid were purchased from Sigma-Aldrich (St. Louis, MO, USA). Ultra-pure water was produced by a Milli-Q system (Millipore, Bedford, MA, USA).

The certified standard solutions were purchased from Romer Labs Diagnostic GmbH (Tulln, Austria). A composite standard working solution of all of the mycotoxins was prepared by dissolving appropriate volumes of each compound in a mobile phase mixture, A:B, 50:50 *v:v* (H_2_O (A) and MeOH (B), both containing 5 mmol·L^−1^ ammonium formate and 0.3% (*v/v*) formic acid). Stock solutions were then diluted with the mobile phase mixture, in order to obtain the appropriate working solutions for the calibration. All solutions were stored at –20 °C in amber glass vials and darkness before use.

### 3.2. Samples

The investigated food samples were obtained from the stock supplied by the “2012–2014 Italian Total Diet Study” [20]. The optimization and validation was conducted on those samples of the food category “Cereals, cereal products, and substitutes” which, in turn, was composed by nine subcategories as follows: “bread”; “pasta”; “pizza”; “rice”; “wheat, other cereals, and flours”; “breakfast cereals”; “biscuits”; “savory fine bakery products”; and “cakes and sweet snacks”. Each subcategory represented a composite core food, which was obtained by pooling a number (up to eight) different “individual samples”, selected according to market share and processing (packed food), origin, and species (fresh food). Each individual sample was, in turn, formed by the combination of a fixed number of “elementary samples” (from 16 to 32) that belonged to the established sampling program conducted within the Italian territory. In some cases the individual samples were prepared and cooked according to normal consumer practices (e.g., pasta and rice were boiled) and then were freeze-dried to enable long-term storage for the study purpose. 

The sampling program considered the collection of elementary samples from the four main geographical areas in Italy (northeast, northwest, center, and south), thus, after pooling the samples of nine subcategories representative of four geographical areas, the “Cereals, cereal products, and substitutes” food category summed up a grand total of 36 samples.

As for the method optimization, a suitable cereal based sample was prepared by combining extra material obtained from the preparation of the TDS samples. The validation was performed on six model matrices (namely bread, pasta, rice, wheat, breakfast cereal, and bakery products) considered as representative of the nine subcategories.

The analytical method was finally applied to all the 36 samples of the “Cereals, cereal products, and substitutes” category, as obtained by the “2012–2014 Italian Total Diet Study” [20], for the multi-mycotoxin determination. Water loss as a consequence of freeze-drying was measured and fresh/dry weight ratios calculated.

### 3.3. Sample Preparation

For all of the tested samples one gram (1.0 g ± 0.1 g) of freeze-dried sample was accurately weighed into a 15 mL centrifuge tube. Samples were extracted by shaking with 8 mL of AcCN:H_2_O (80:20) 1% HCOOH on a mechanical wrist shaker for 30 min. Extracted samples were centrifuged at 10,000 rpm for 10 min. Four milliliters of supernatant were dried under a stream of nitrogen, redissolved with 400 μL of injection solution (mobile phase A:B, 50:50), and then centrifuged at 10,000 rpm for 10 min. Prior to injection on the LC-MS/MS system, the samples were filtered through a 4 mm, 0.2 μm polytetrafluoroethylene (PTFE) syringe filter (Sartorius, Göttingen, Germany.

### 3.4. LC-MS/MS Analysis

A Waters UPLC system (Waters, Milford, MA, USA) was used to perform a reverse phase chromatography separation of the selected mycotoxins. The separation was achieved by a Kinetex Biphenyl column (50 mm × 3 mm i.d., 2.6 μm particle size) preceded by a SecurityGuard^TM^ ULTRA Holder pre-column, both supplied by Phenomenex (Phenomenex, Torrance, CA, USA). The mobile phase was a time-programmed gradient using H_2_O (eluent A) and MeOH (eluent B), both containing 5 mmol L^−1^ ammonium formate and 0.3% (*v/v*) formic acid. Gradient elution was started isocratically with 95% A for 1 min. Then, B was linearly increased to 100% within 7.5 min and kept constant for 2 min. Finally, B was decreased linearly to 5% in 1.0 min and equilibrated for 5 min. The flow rate was set at 300 μL min^−1^.

The LC system was coupled with a Waters Quattro Premier XE TQ mass spectrometer (Waters, Manchester, UK) equipped with an ESI source operating in positive ionization mode (ESI+). ESI-MS/MS was performed in multiple reaction monitoring (MRM). The MassLynx v4.1 (Waters, Milford, MA, USA) was used in order to control the UPLC-MS/MS system. Capillary voltage, source temperature, desolvation gas flow rate, and its temperature were set at 3 kV, 120 °C, 600 L h^−1^, and 350 °C, respectively. Collision-induced dissociation was performed using argon as collision gas at a pressure of 3.5 × 10^−3^ mbar in the collision cell. Cone voltage (CV) and collision energy (CE) values were optimized for each precursor ion and different product ions. For each compound, at least one precursor and two product ions for both identification and quantification purposes were identified, selecting the most abundant product ion for quantification and the second one for confirmation purposes. The precursor ion and the optimized MS/MS parameters (cone voltage and collision energy) for each analyte are summarized in Table 1.

### 3.5. Method Performance

Linearity, apparent recovery, matrix effect, and recovery of extraction were evaluated by preparing a set of three calibration curves prepared (i) in neat solvent; (ii) by spiking the extract of a blank sample (spiked extract curve); and (iii) by spiking a blank sample before the extraction step (spiked sample curve).

The matrix-matched calibration curves (spiked extract and spiked samples curves) were prepared for each validated matrix. The described set of calibration curves was processed in replicates (*n* = 6) for repeatability evaluation. The spiked samples used for the spiked calibration curve were also used as spiking experiments for the assessment of trueness and precision.

Limits of detection (LoDs) based on a signal-to-noise (S/N) ratio of 3:1, and LoQs on a S/N ratio of 6:1, were calculated by injecting neat solvent standard solutions at different concentration levels. The calculated LoQs were than verified and validated on the different analyzed matrices, being one of the selected concentration levels of the replicated (*n* = 6) spiking experiments.

#### 3.5.1. Linearity

Linearity was evaluated by preparing a six-concentration levels calibration curve. The concentration range for each mycotoxin is reported in Table 5. The spiked sample curves were used for quantification, each working day the calibration curve was constructed in duplicate and the average values were considered. The matrix-matched calibration curves were prepared for all of the six validated matrices.

#### 3.5.2. Apparent Recovery, Matrix Effect, Recovery of Extraction

The evaluation of apparent recovery, signal suppression/enhancement due to matrix effects and extraction recovery were calculated from the six points calibration curves as described in Section 3.5.1, as follows [15]:

(1) RA  (%)=100×slopespiked sampleslopeneat solvent

(2) SE (%)=100×slopespiked exrtactslopeneat solvent

(3) RE  (%)=100×RASSE

where *slope_spiked sample_*, *slope_neat solvent_*, and *slope_spiked extract_* represent the values of the gradient of the three calibration curves obtained by plotting peak areas: (1) of the spiked samples; (2) of the neat standards; and (3) of the spiked extracts versus the analyte concentration. Each curve was run in duplicate and each spiking experiment was replicated six times to assess repeatability.

Trueness was assessed following EURACHEM criteria [26] by performing replicated spiking experiments (*n* = 6) at the LoQ levels for each of the model matrix for all of the tested mycotoxins. The average values of recovery were calculated and the standard deviations of repeatability from the six replicates were taken as a measure of the precision of the method.

With respect to the identification requirements for mass spectrometric detection of mycotoxins, the retention times of the analytes in the sample extract were checked to correspond to that of the average of the calibration standards measured in the same sequence with a tolerance of ±0.1 min; the ion ratio (defined as the response of the peak with the lower area divided by the response of the peak with the higher area) was checked to be within ±30% (relative) to that obtained from the average of the calibration standards from the same sequence.

## 4. Conclusions

The proposed method represents an example of multi-mycotoxin determination in composite cereal-based samples fit for the purpose of a TDS study. The “Cereals, cereal products, and substitutes” was the category of reference in which nine kinds of subcategories (i.e., “bread”; “pasta”; “pizza”; “rice”; “wheat, other cereals, and flours”; “breakfast cereals”; “biscuits”; “savory fine bakery products”; and “cakes and sweet snacks”) were included. The number of publications available for LC-MS/MS techniques around food and feed subject matter are extensive [17,18,23,24], and all kind of references criteria are defined on the basis of “real life” scenarios very much depending on either the specific group (or number) of toxins or combination (and kinds) of matrices. The need to reach low levels of LoQ and satisfactory performances for all the considered mycotoxins was successfully achieved by means of a robust matrix matched validation experiments performed on a selected model matrix samples. The lack of validation criteria/acceptability criteria specifically addressed to mycotoxins in the context of LC-MS method, pushed us to assess the outputs on the basis of the general criteria of precision and recovery [27] and the obtained results are encouraging in terms of acceptable and satisfactory performances to be considered in the context of a multi-toxin analytical method for complex matrices. However, the dynamism in terms of extension to emerging toxins, MS method development, and the setting of new maximum limits compels the researchers to constantly improve method performances, especially in those remarkable cases, such as baby foods, for which the requested LoQs represent a real challenge.

## Figures and Tables

**Table 1:** Optimized MS/MS parameters for the analyzed mycotoxins.

Mycotoxin	Retention Time (min)	Precursor Ion (m/z)	Product Ions (m/z) ^a^	Collision Energy (V) ^a^	Cone Voltage (V)
AFB1	8.15	313.2 [M + H]^+^	285.3/241	21/35	45
OTA	7.35	404.1 [M + H]^+^	238.8/358.1	25/14	25
DON	3.89	297.3 [M + H]^+^	203.5/249.5	15/10	22
FB1	5.55	723.3 [M + H]^+^	335.1/353.5	40/30	50
T-2	6.24	489.6 [M + Na]^+^	245.1/327.1	30/20	30
HT-2	5.77	447.2 [M + NH4]^+^	285.1/345.1	15/10	30
ZEA	6.79	319.3 [M + H]^+^	282.9/301.1	10/10	22

^a^ Numerical value are given in the order quantifier/qualifier.

**Table 2:** Solvent mixtures tested for extraction step optimization, recovery factors (%) and RSD of repeatability (%). Results for aflatoxin B1 (AFB1) and OTA were grouped, as well as for FB1, DON, T-2, and HT-2 toxins and ZEA.

Extraction Solvent	Recovery Factors (RSD_r_^a^)	
	AFB1, OTA	FB1, DON, T-2 and HT-2, ZEA
CH_3_OH:H_2_O 80:20	75 (20)	30 (19)
CH_3_CN:H_2_O:CH_3_COOH 79:20:1	65 (15)	80 (15)
H_2_O 4mL + EtOH 8mL	25 (20)	80 (19)

^a^ Relative standard deviation, calculated on six replicates processed under repeatability operating conditions.

**Table 3:** Determination coefficient (*R*^2^) obtained for the targeted mycotoxins in the six validated cereal based matrices.

Mycotoxin	*R* ^2^						
	Neat Solvent	Bread	Pasta	Rice	Wheat	Bakery Products	Breakfast Cereal
AFB1	0.9995	0.9956	0.9997	0.9901	0.9913	0.9974	0.9995
OTA	0.9976	0.9958	0.9995	0.9994	0.9903	0.9965	0.9989
FB1	0.9975	0.9982	0.9964	0.9968	0.9982	0.9993	0.9969
DON	0.9941	0.9993	0.9997	0.9993	0.9978	0.9927	0.9914
T-2	0.9937	0.9983	0.9925	0.9929	0.9969	0.9996	0.9906
HT-2	0.9973	0.9909	0.9927	0.9952	0.9919	0.9935	0.9950
ZEA	0.9926	0.9963	0.9937	0.9939	0.9902	0.9942	0.9902

**Table 4:** Apparent recovery, signal suppression/enhancement, extraction recovery, and relative standard deviation of repeatability of R_A_ obtained for all of the selected mycotoxins and for each validated matrix, together with the validated LoQs.

Matrix		AFB1	OTA	FB1	DON	T-2	HT-2	ZEA
Bread	LoQ (μg/kg)	0.13	0.8	16	20	8	20	3.2
	R_A_ (%)	61	64	84	62	65	62	65
	SSE (%)	83	98	113	61	55	63	75
	R_E_ (%)	73	65	74	100	119	99	87
	RSD_r_ (%)	14	10	15	18	17	15	20
Pasta	LoQ (μg/kg)	0.06	0.4	8	20	4	20	1.6
	R_A_ (%)	61	79	116	69	75	79	75
	SSE (%)	52	102	120	66	96	109	83
	R_E_ (%)	117	78	96	105	81	73	90
	RSD_r_ (%)	11	12	11	12	16	15	16
Rice	LoQ (μg/kg)	0.06	0.4	8	20	4	20	1.6
	R_A_ (%)	88	79	93	70	65	63	78
	SSE (%)	105	102	101	107	74	64	75
	R_E_ (%)	83	78	92	65	88	98	104
	RSD_r_ (%)	11	10	10	15	14	13	15
Wheat	LoQ (μg/kg)	0.06	0.4	8	20	4	20	1.6
	R_A_ (%)	69	76	122	66	61	66	65
	SSE (%)	73	92	123	71	64	73	65
	R_E_ (%)	94	83	99	92	95	90	101
	RSD_r_ (%)	12	10	12	15	15	15	15
Backery products	LoQ (μg/kg)	0.13	0.8	16	20	8	20	3.2
	R_A_ (%)	66	85	76	67	65	63	64
	SSE (%)	84	108	123	61	64	55	74
	R_E_ (%)	79	79	61	109	102	116	87
	RSD_r_ (%)	13	10	16	18	15	15	18
Breakfast cereals	LoQ (μg/kg)	0.13	0.8	16	20	8	20	3.2
	R_A_ (%)	65	68	63	66	69	69	68
	SSE (%)	59	95	123	74	72	68	71
	R_E_ (%)	110	71	52	89	96	101	96
	RSD_r_ (%)	16	6	15	14	19	11	20

**Table 5:** Concentration levels prepared for the selected mycotoxins.

Mycotoxin	Concentration Ranges	
	ng/mL	ng/g
AFB1	0.075–2.000	0.06–1.60
OTA	0.5–12.5	0.4–10.0
FB1	10–250	8–200
DON	25–625	20–500
T-2	5–125	4–100
HT-2	25–625	20–500
ZEA	2–50	1.6–40
